# Chloroplast Microsatellite Diversity in *Phaseolus vulgaris*

**DOI:** 10.3389/fpls.2012.00312

**Published:** 2013-01-22

**Authors:** F. Desiderio, E. Bitocchi, E. Bellucci, D. Rau, M. Rodriguez, G. Attene, R. Papa, L. Nanni

**Affiliations:** ^1^Dipartimento di Scienze Agrarie, Alimentari ed Ambientali, Università Politecnica delle MarcheAncona, Italy; ^2^Dipartimento di Agraria, Università degli Studi di SassariSassari, Italy; ^3^Centro per la Conservazione e la Valorizzazione della Biodiversità Vegetale, Università degli Studi di SassariSurigheddu, Alghero, Italy; ^4^Cereal Research Centre, Consiglio per la Ricerca e Sperimentazione in AgricolturaFoggia, Italy

**Keywords:** *Phaseolus*, crop evolution, cpSSR, recombination, population structure, speciation, introgression

## Abstract

Evolutionary studies that are aimed at defining the processes behind the present level and organization of crop genetic diversity represent the fundamental bases for biodiversity conservation and use. A Mesoamerican origin of the common bean *Phaseolus vulgaris* was recently suggested through analysis of nucleotide polymorphism at the nuclear level. Here, we have used chloroplast microsatellites to investigate the origin of the common bean, on the basis of the specific characteristics of these markers (no recombination, haploid genome, uniparental inheritance), to validate these recent findings. Indeed, comparisons of the results obtained through analysis of nuclear and cytoplasmic DNA should allow the resolution of some of the contrasting information available on the evolutionary processes. The main outcomes of the present study are: (i) confirmation at the chloroplast level of the results obtained through nuclear data, further supporting the Mesoamerican origin of *P. vulgaris*, with central Mexico representing the cradle of its diversity; (ii) identification of a putative ancestral plastidial genome, which is characteristic of a group of accessions distributed from central Mexico to Peru, but which have not been highlighted beforehand through analyses at the nuclear level. Finally, the present study suggests that when a single species is analyzed, there is the need to take into account the complexity of the relationships between *P. vulgaris* and its closely related and partially intercrossable species *P. coccineus* and *P. dumosus*. Thus, the present study stresses the importance for the investigation of the speciation processes of these taxa through comparisons of both plastidial and nuclear variability. This knowledge will be fundamental not only from an evolutionary point of view, but also to put *P. coccineus* and *P. dumosus* germplasm to better use as a source of useful diversity for *P. vulgaris* breeding.

## Introduction

The wild forms of the common bean *Phaseolus vulgaris* grow across a wide geographic area of the Americas, from northern Mexico to northwestern Argentina (Toro et al., 1990). Morphological, biochemical, and molecular data have indicated that the wild populations from Mexico, Central America, and Colombia differ from those of southern Peru, Bolivia, and Argentina (Gepts et al., 1986; Delgado-Salinas et al., 1988; Koenig and Gepts, 1989; Gepts and Debouck, 1991; Becerra-Velásquez and Gepts, 1994; Papa and Gepts, 2003; Angioi et al., 2009a; Kwak and Gepts, 2009; Rossi et al., 2009). Indeed, these two groups represent two geographically distinct and isolated gene pools (Mesoamerica and Andes, respectively) that were already present before domestication of the common bean (for reviews, see Papa et al., 2006; Bitocchi et al., 2012, 2013). This complex scenario is further characterized by the presence within the wild forms of a third gene pool that is characteristic of a restricted area in northern Peru and Ecuador (Debouck et al., 1993). Along with accessions from the two main gene pools, wild populations collected in this restricted area have been analyzed according to a portion of the gene encoding for the seed-storage protein phaseolin (Kami et al., 1995). This study showed that the “Inca” phaseolin type I is not present in Central and South America. Moreover, this phaseolin appears to be ancestral to the other phaseolin sequences of *P. vulgaris*, suggesting that the northern Peru and Ecuador populations were those from which the common bean originated and subsequently spread into Central and South America (Kami et al., 1995). This hypothesis was the most credited until the study of Bitocchi et al. (2012) that analyzed the genetic diversity at five nuclear gene fragments across a wide sample of wild *P. vulgaris* accessions, where they showed that the wild forms of *P. vulgaris* originated in Mesoamerica, and most likely in central Mexico. This study also indicated that both the Andean and the northern Peru and Ecuador gene pools originated through different migration events from central Mexico. This conclusion was suggested by the evidence of a bottleneck that occurred in the Andes prior to domestication (Rossi et al., 2009; Nanni et al., 2011; Bitocchi et al., 2012) and to the presence of high genetic structure in Mesoamerica (Bitocchi et al., 2012), with the different genetic groups identified having diverse relationships with the wild populations from northern Peru and Ecuador and from the Andes.

Chloroplast microsatellite (cpSSR) markers are widely used in population genetics and evolutionary studies of plants (for review, see Provan et al., 2001). Due to their specific characteristics, which include a haploid and non-recombinant genome and uniparental inheritance, they have become very useful tools to investigate different evolutionary processes. These include, e.g., historical bottlenecks, founder effects, identification of progenitors of cultivated species, and the role of introgression in crop evolution (for review, see Provan et al., 2001).

In the present study, we used a set of cpSSRs to analyze a wide sample of wild *P. vulgaris* accessions from the Americas. These cpSSRs have been demonstrated to be very useful to study the diversity and evolution of several legume species, and in particular of *P. vulgaris* and *P. coccineus* (Angioi et al., 2009a,b, 2010). The main aim was to investigate the origin of the common bean and to compare the results with those obtained by analyses based on nuclear nucleotide diversity (Bitocchi et al., 2012). Indeed, at the nuclear level, recombination might have affected the data obtained, although to reduce this problem, fragments of a few hundreds of base pairs were used. Thus, the comparison and combination of nuclear and plastidial polymorphism analyses should give complementary insights into the evolutionary history of the common bean, especially considering that such analyses can often provide contrasting information on evolutionary processes (Birky, 1988; McCauley, 1995; Ennos et al., 1999; Provan et al., 1999; Weising and Gardner, 1999; Ishii et al., 2001; Lira et al., 2003; Ueno et al., 2005).

Finally, cpSSR genotyping of a smaller set of *P. coccineus* accessions was carried out, with the aim being to gain information about the evolutionary relationship between *P. coccineus* and *P. vulgaris*.

## Materials and Methods

### Plant materials

**Figure 1:**
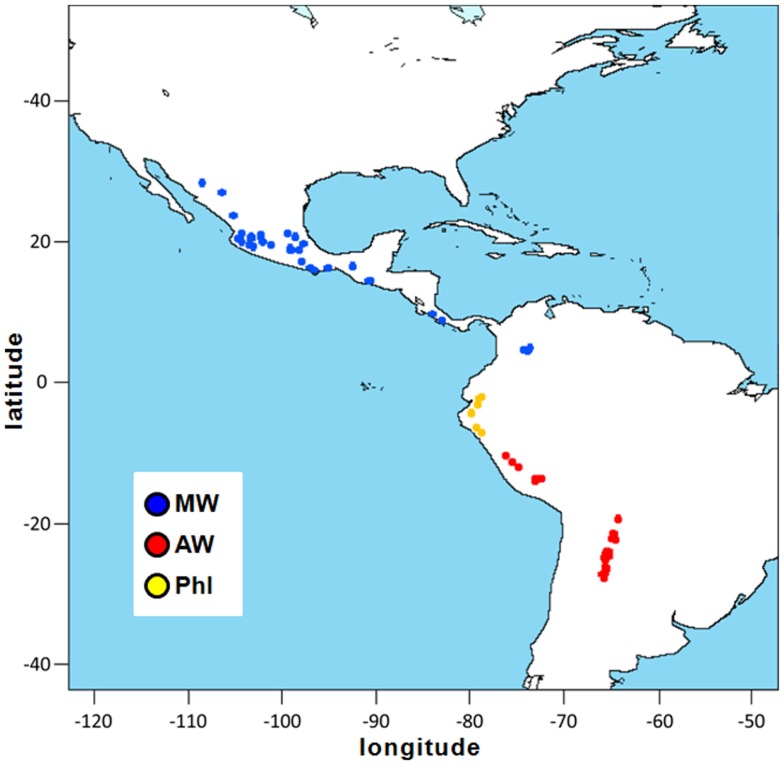
**Geographical distribution of the *P. vulgaris* accessions used in the present study**. Latitude and longitude are expressed in the Universal Transverse Mercator system. MW, Mesoamerican wild; AW, Andean wild; PhI, northern Peru and Ecuador.

A total of 109 wild accessions of *P. vulgaris* were analyzed in the present study. These materials encompassed the entire geographical distribution of this species, from northern Mexico to northwestern Argentina, and included seven wild accessions from northern Peru and Ecuador that are characterized by the ancestral phaseolin type I (Debouck et al., 1993; Kami et al., 1995). The geographical distribution of these common bean accessions is shown in Figure 1. Ten wild accessions of *P. coccineus* were also included. Each accession is represented by an individual plant genotype. A complete list of the accessions studied, along with their “passport” information, is given in Table A1 in Appendix.

The seeds were provided by the United States Department of Agriculture (USDA) Western Regional Plant Introduction Station in the USA, the International Center of Tropical Agriculture (CIAT) in Colombia, and the Laboratory of Plant Genetics (D3A) at the Polytechnic University of Marche (UNIVPM) in Italy. Most of these accessions had already been characterized using different types of molecular markers, such as amplified fragment length polymorphism (AFLP; Rossi et al., 2009) and nucleotide data (Nanni et al., 2011; Bitocchi et al., 2012). Moreover a small subset of accessions (15 wild *P. vulgaris*, eight wild *P. coccineus*) were analyzed previously by Angioi et al. (2009a) with the same set of cpSSRs.

### PCR and cpSSR genotyping

Genomic DNA was extracted from each accession from young leaf tissue of a single, greenhouse-grown plant, using the miniprep extraction method (Doyle and Doyle, 1987). A total of 17 cpSSRs derived from the literature (Weising and Gardner, 1999; Chung and Staub, 2003; Angioi et al., 2009a) were used for the genetic characterization of the whole sample. One of the two SSR primers was end-labeled with a phosphoramidite fluorescent dyes, 6-FAM or HEX. A list of the cpSSRs used in this study is given Table A2 in Appendix. The amplifications were conducted using a Perkin-Elmer 9700 Thermal Cycler (PE Applied Biosystems) in a total volume of 25 μl, which contained 25 ng template DNA, 10 pmol of each primer, 200 μM dNTPs, 1× *Taq* polymerase buffer, 2.5 mM MgCl_2_ and 1 U *Taq* polymerase (Promega). The PCR conditions were as reported in Table A2 in Appendix. Multiplex PCRs were performed (including two primer pairs that were differently end-labeled, with amplification of SSRs of different sizes under the same amplification conditions). SSR genotyping was carried out using the ABI PRISM 3100-Avant Genetic Analyzer with the GeneScan 7.0 analysis software (PE Applied Biosystems).

### Genetic diversity analysis

The percentage of polymorphic loci, the average number of observed alleles per locus (Na), the effective number of alleles per locus (Ne; Kimura and Crow, 1964), the number of private alleles (Np), and the expected heterozygosity (He; Nei, 1978) estimates based on allele frequencies, were computed using the Arlequin software, version 3.5 (Excoffier and Lischer, 2010). The whole sample, and the following partitions of the accessions were considered for these analyses: *P. coccineus*; *P. vulgaris*; and within the common bean sample according to the gene pool, the Andean wild (AW), Mesoamerican wild (MW), and northern Peru and Ecuador (PhI) populations.

The differences between the AW and MW populations for the genetic diversity estimates (Ne and He) were tested using Wilcoxon signed-ranks non-parametric test for two groups, arranged for paired observations (i.e., one pair of estimates for each locus; Wilcoxon, 1945; Sokal and Rohlf, 1995).

An *ad hoc* statistic (Δ*H*) was used to compare the diversity between the two main gene pools (AW, MW); this estimate measures the loss of diversity of one population compared to another, and it was originally proposed by Vigouroux et al. (2002): Δ*H* = 1 − (He_POP1_/He_POP2_), where POP1 refers to the population that shows the lower level of genetic diversity (He) compared to the other population (POP2).

### Principal component analysis

Using the JMP software, version 8 (SAS Institute, Inc., 2008), principal component analysis (PCA) was performed from allele frequencies. The same analysis was carried out also to investigate the genetic relationships among the *P. vulgaris* accessions.

### Population structure analysis

A Bayesian model-based approach that was implemented in the Bayesian analysis of population structure (BAPS) software, version 5.3 (Corander et al., 2003), was used to infer the hidden genetic population structure of the whole sample (109 *P. vulgaris* and 10 *P. coccineus* accessions), and thus to assign the genotypes into genetically structured groups/populations (K). A spatial genetic mixture analysis was conducted (Corander et al., 2008). This method uses a Markov chain Monte Carlo simulation approach to group samples into variable user-defined numbers (K) of clusters. The best partition of populations into K clusters is identified as the one with the highest marginal log-likelihood. We carried out 10 repetitions of the algorithm for each K ranging between 2 and 20.

The genetic diversity statistics described above were also computed for the genetic groups highlighted by the BAPS analysis (hereafter referred to as clusters). The differences between the clusters identified according to the genetic diversity estimates (Ne, He) were tested using the Wilcoxon signed-ranks non-parametric test for two groups, arranged for paired observations (Wilcoxon, 1945; Sokal and Rohlf, 1995), and the Bonferroni correction for multiple comparisons.

### Divergence between populations

The divergence among the *P. coccineus* and *P. vulgaris* populations defined *a priori* according to the gene pools (AW, MW, PhI) were estimated as *F*_ST_ (Weir and Cockerham, 1984), *D* (Jost, 2008), and *R*_ST_ (Slatkin, 1995). In contrast to *F*_ST_ and *D*, *R*_ST_ contains information not only about the frequency with which particular alleles occur, but also on the evolutionary distance between them, inasmuch as it is measured as the expected squared difference in repeat numbers between alleles. For this reason, it is intended to take advantage of this additional information to provide greater insight into the patterns of relationships among populations (for review, see Holsinger and Weir, 2009). These correspond to the infinite allele and the step-wise mutation models. The significance of the estimates was obtained through permutation tests, using 10,000 permutations. The same divergence estimates were also computed for clusters identified by BAPS analysis. The Arlequin software, version 3.5 (Excoffier and Lischer, 2010), was used.

### Comparison of results based on cpSSR data with those obtained using nucleotide data

The sequences of five gene regions (from 500 to 900 bp) for 71 accessions were available from Bitocchi et al. (2012). These five gene fragments include four legume anchor (Leg) markers, developed by Hougaard et al. (2008), and one gene fragment, *PvSHP1*, developed by Nanni et al. (2011); *PvSHP1* is a homolog of the SHATTERPROOF (SHP1) gene, which is involved in the control of fruit shattering in *Arabidopsis thaliana*. These data allowed a comparison of the data from the population structure analyses carried out using cpSSRs and nuclear sequences. Thus, for the 71 accessions shared between this study and that of Bitocchi et al. (2012), a population structure analysis was carried out using both the cpSSRs and the nucleotide data. For the nucleotide data, the procedures were as described in Bitocchi et al. (2012), while for the cpSSRs, the procedures were the same as reported in the above section.

To compare the geographical distributions of the clusters identified through the cpSSR and nucleotide data, spatial interpolation of membership coefficients (*q*) was performed according to the kriging method, with each of the clusters identified by population structure analysis, which was implemented in the R packages spatial (http://www.r-project.org/). In the case of the cpSSRs, due to the non-recombinant nature of these markers, which does not allow admixture, the membership coefficients were represented by one or zero (i.e., membership or non-membership to one cluster); thus, the interpolation for plastidial data represents an approximation.

The association between the results obtained by the BAPS analyses carried out with the cpSSR and nucleotide data was tested by analysis of contingency tables with the likelihood ratio chisquared (χ^2^) test, which was performed using the JMP 8.0 software (SAS Institute, Inc., 2008).

## Results

Each of the primer pairs produced a single and clear amplification, and all of the 17 loci studied were polymorphic considering the whole analyzed sample. The size of the amplification products ranged from 79 bp (ccmp3) to 378 bp (ccSSR19). Overall, the number of alleles per locus (Na) ranged from two (cp2) to 12 (ccSSR20); in parallel the same two markers showed the lowest and the highest genetic diversity, He = 0.13 and He = 0.85, respectively (Table A3 in Appendix).

Considering the *P. coccineus* sample, six out of the 17 loci were monomorphic. For the polymorphic loci, Na ranged from two (cp2, ccSSR2, ccSSR4, ccSSR12, and ccSSR16) to six (ccSSR20). One locus (cp2) was monomorphic in the *P. vulgaris* sample. For the remaining 16 loci, Na ranged from two (cp3 and ccSSR12) to 11 (ccSSR20). The highest level of genetic diversity was detected for the ccSSR20 locus, as an He of 0.84 for both *P. vulgaris* and *P. coccineus* (Table A3 in Appendix).

### Genetic diversity analysis

Genetic diversity estimates were computed considering the whole sample and the following major subdivisions: different species (*P. vulgaris*, *P. coccineus*) and within the *P. vulgaris* Andean (AW), Mesoamerican (MW), and northern Peru and Ecuador accession (PhI) populations.

As showed in Table 1, the common bean was characterized by a higher level of genetic diversity (Na, Ne, Np, and He) than *P. coccineus*. However, the large difference between the size of the two samples suggests caution in the consideration of these estimates.

**Table 1:** **Genetic diversity estimates computed for all of the 17 cpSSR loci considering the whole sample, the *P. vulgaris* and *P. coccineus* samples, and the three *P. vulgaris* populations defined according to the gene pools**.

Accession	*N*	% polymorphic loci	Na	Ne	Np	Np (freq. ≥ 0.05)	He
All	119	100	5.1	2.6	na	na	0.54
*P. vulgaris*	109	94.1	4.4	2.5	45	29	0.51
*P. coccineus*	10	64.7	2.4	1.8	12	12	0.36
*P. vulgaris* populations							
MW	55	88.2	3.9	2.5	7	3	0.54
AW	47	82.4	3.2	1.9	4	3	0.40
PhI	7	82.4	2.5	2.2	3	3	0.49

*N, sample size; Na, mean number of observed alleles per locus; Ne, mean effective number of alleles per locus; Np, number of private alleles; Np (freq. ≥ 0.05), number of private alleles with frequency higher than 0.05; He, expected heterozygosity; MW, Mesoamerican wild; AW, Andean wild; PhI, northern Peru and Ecuador; na, not applicable*.

Among the three *P. vulgaris* populations, the MW accessions showed the highest genetic diversity for all of the parameters (Table 1). In particular, considering the populations that represent the two major common bean gene pools (Mesoamerican and Andean), the MW showed a higher level of genetic diversity (Ne = 2.5 and He = 0.54) compared to the AW accessions (Ne = 1.9 and He = 0.40; Table 1). This difference was significant for both the genetic diversity estimates Ne and He (*P* < 0.02; Wilcoxon signed-ranks non-parametric test for two groups, arranged for paired observations). There was a 26% reduction in genetic diversity (Δ*H*) of the AW population compared to the MW population.

### Principal component analysis

**Figure 2:**
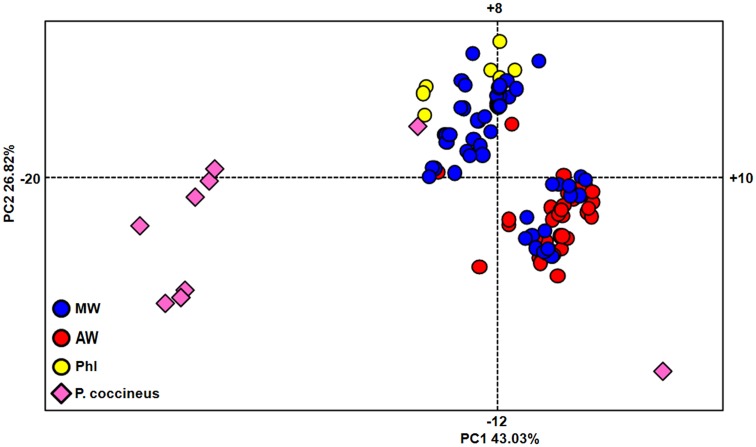
**Genetic relationships within the whole set of accessions, as determined by principal component analysis**. MW, Mesoamerican wild; AW, Andean wild; PhI, northern Peru and Ecuador.

The relationships among all of the individuals considered, including both the *P. vulgaris* and *P. coccineus* accessions, were investigated by PCA (Figure 2). The first (PC1) and second (PC2) principal components explain 43.03 and 26.82%, respectively. Three main groups were identified by this analysis, one including eight wild *P. coccineus* accessions, one including all of the seven PhI, two WA, and 39 WM accessions and one *P. coccineus* accession, and the remaining 45 WA and 16 WM accessions, and even if more distant, one *P. coccineus* accessions.

**Figure 3:**
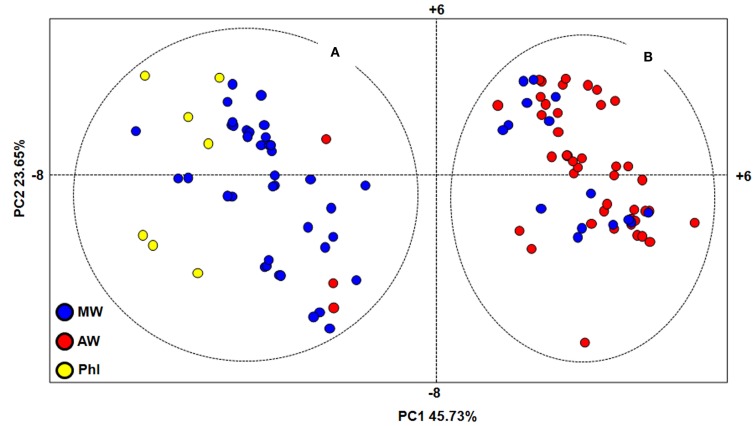
**Genetic relationships within the *P. vulgaris* accessions, as assessed by principal component analysis**. MW, Mesoamerican wild; AW, Andean wild; PhI, northern Peru and Ecuador; **(A,B)**, major groups identified by PCA analysis.

Principal component analysis was also performed to investigate the genetic relationships among the *P. vulgaris* accessions (Figure 3). The first (PC1) and second (PC2) principal components explain 45.73 and 23.65%, respectively. This analysis identified two major groups, as A and B (Figure 3). The majority of the MW accessions (73%; including five of the six Colombian accessions) belonged to group A, along with three AW accessions from northern Argentina (Salta and Tucumán Provinces) and all of the seven PhI accessions. Group B included almost all of the AW accessions (94%) and 15 MW accessions, 14 of which were from central Mexico, and only one from Colombia.

### Population structure

**Figure 4:**
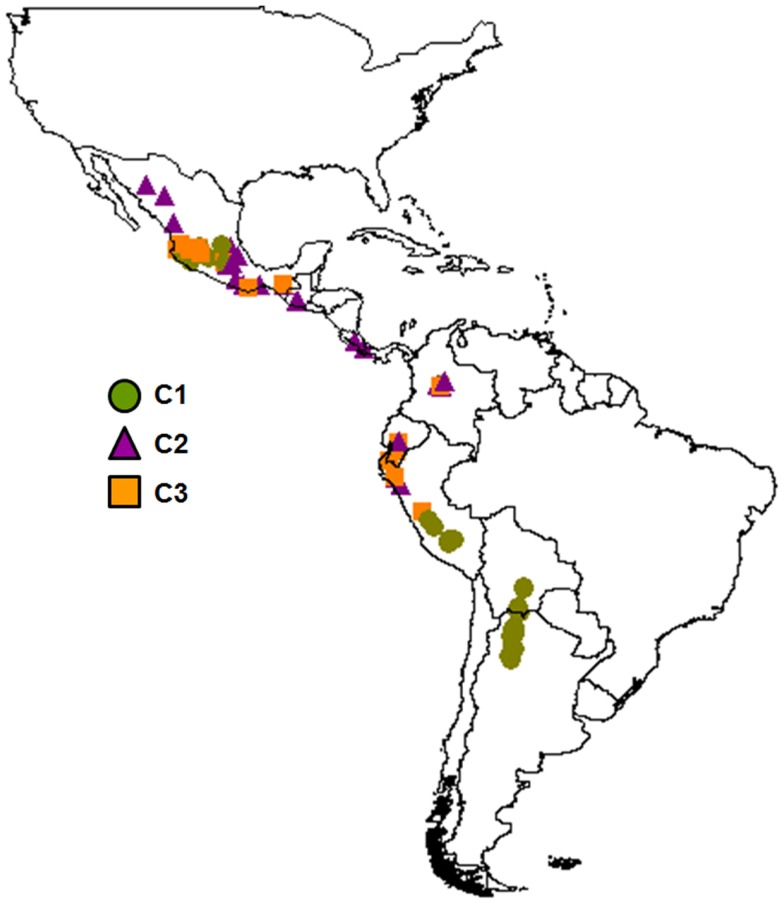
**Geographical distribution of the *P. vulgaris* accessions based on the BAPS cluster membership**.

The population structure analysis identified four different clusters (*C*1, *C*2, *C*3, *C*4) as the best partition of the whole sample (all of the 10 best marginal log-likelihood values were for *K* = 4, with the highest of −1,996.54; Table 2). Cluster *C*1 was characterized by almost all of the AW accessions (98%) and 13 MW accessions from Central Mexico. Cluster *C*2 included 21 MW and three PhI accessions, along with two *P. coccineus* genotypes. There were accessions from all of the three common bean populations in cluster *C*3 (4, 1, 21 for the PhI, AW, MW populations, respectively), while cluster *C*4 was exclusive to the remaining eight *P. coccineus* accessions. The geographical distribution of the *P. vulgaris* accessions based on the BAPS cluster membership is showed in Figure 4.

**Table 2:** **Distribution of the accessions into the four cpSSR clusters (*C*1, *C*2, *C*3, *C*4) identified by the BAPS analysis**.

Accession	Cluster			
	*C*1	*C*2	*C*3	*C*4
MW	13	21	21	–
AW	46	–	1	–
PhI	–	3	4	–
*P. coccineus*	–	2	–	8
Overall	59	26	26	8

*MW, Mesoamerican wild; AW, Andean wild; PhI, northern Peru and Ecuador*.

The genetic diversity estimates for the BAPS clusters are showed in Table 3. The three clusters characteristic of *P. vulgaris* accessions (*C*1, *C*2, *C*3) showed similar levels of genetic diversity (Ne = 2.0, 2.1, 1.8, and He = 0.42, 0.45, 0.36, for *C*1, *C*2, *C*3, respectively). Cluster *C*4 showed the lowest Ne (1.6) and He (0.29) estimates. However, there were no significant differences in the levels of genetic diversity between these four clusters (Wilcoxon signed-ranks non-parametric tests, after Bonferroni correction).

**Table 3:** **Genetic diversity estimates computed for the 17 cpSSRs considering the four clusters (*C*1, *C*2, *C*3, and *C*4) identified by BAPS analysis**.

Cluster	*N*	% polymorphic loci	Na	Ne	Np	Np (freq. ≥ 0.05)	He
*C*1	59	88.2	3.4	2.0	6	5	0.42
*C*2	26	88.2	3.2	2.1	3	0	0.45
*C*3	26	88.2	3.1	1.8	7	3	0.36
*C*4	8	52.9	1.9	1.6	10	10	0.29

*N, sample size; Na, mean number of observed alleles per locus; Ne, mean effective number of alleles per locus; Np, number of private alleles; Np (freq. ≥ 0.05), number of private alleles with frequency higher than 0.05; He, expected heterozygosity*.

### Divergence between populations

The genetic divergence between the *P. vulgaris* populations (MW, AW, PhI) and the *P. coccineus* accessions was estimated as *F*_ST_, *D*, and *R*_ST_. The *F*_ST_ and *D* estimates were very similar, as expected for populations that have a very low number of unique alleles (Whitlock, 2011), and thus only the *F*_ST_ data are shown. The lowest, and non-significant, differentiation was between the PhI and MW populations (*F*_ST_ = 0.08; *R*_ST_ = 0.12; both non-significant; Table 4). Considering the comparisons among the *P. vulgaris* populations, the divergence between AW and PhI (*F*_ST_ = 0.21; *R*_ST_ = 0.70; both significant *P* ≤ 0.001) was greater than that between AW and MW (*F*_ST_ = 0.13; *R*_ST_ = 0.24; both significant *P* ≤ 0.01). The highest values of *F*_ST_ were those in the comparisons with the *P. coccineus* population; however, the MW population showed the lowest levels of differentiation with *P. coccineus* (*F*_ST_ = 0.33; *P* ≤ 0.001) compared to the other *P. vulgaris* populations [*F*_ST(PhI-*P. coccineus*)_ = 0.38, *P* ≤ 0.001; *F*_ST(AW-*P. coccineus*)_ = 0.49, *P* ≤ 0.001; Table 4]. The *R*_ST_ showed a similar trend, with the MW population being less differentiated than *P. coccineus* (*R*_ST_ = 0.58, *P* ≤ 0.001), and PhI [*R*_ST(PhI-*P. coccineus*)_ = 0.60, *P* ≤ 0.001], and AW [*R*_ST(AW-*P. coccineus*)_ = 0.78, *P* ≤ 0.001; Table 4].

**Table 4:** **Genetic divergence (*F*_ST_ and *R*_ST_, below and above the diagonal, respectively) within the *P. vulgaris* populations and with *P. coccineus***.

	MW	AW	PhI	*P. coccineus*
MW	–	0.13*	0.08	0.58**
AW	0.24*	–	0.21**	0.78**
PhI	0.12	0.70**	–	0.60**
*P. coccineus*	0.33**	0.49**	0.38**	–

Significance obtained by 10,000 permutations: **P ≤ 0.001; *P ≤ 0.01.

The same divergence estimates were computed considering the four genetic clusters (*C*1, *C*2, *C*3, *C*4) identified by the BAPS analysis (Table 5). All of the divergence estimates (for both *F*_ST_ and *R*_ST_) were significantly different from zero (*P* ≤ 0.001). We observed less differentiation (lower *F*_ST_ and *R*_ST_) among the three clusters predominated by the *P. vulgaris* accessions (*C*1, *C*2, *C*3), than between any of these and *C*4, which was comprised exclusively of *P. coccineus* accessions. When considering these comparisons with the *P. coccineus* cluster (*C*4), the lowest *F*_ST_ was with the *C*2 cluster [*F*_ST(*C*2–*C*4)_ = 0.39]. *R*_ST_ gave a slightly different pattern, with comparisons involving the *C*3 cluster showing the lowest *R*_ST_ (Table 5).

**Table 5:** **Genetic divergence (*F*_ST_ and *R*_ST_, below and above the diagonal, respectively) between the four cpSSR clusters identified by population structure analysis**.

	*C*1	*C*2	*C*3	*C*4
*C*1	–	0.54**	0.37**	0.90**
*C*2	0.26**	–	0.15**	0.81**
*C*3	0.28**	0.37**	–	0.68**
*C*4	0.50**	0.39**	0.56**	–

Significance obtained by 10,000 permutations: **P ≤ 0.001.

### Nucleotide data *versus* cpSSRs

**Figure 5:**
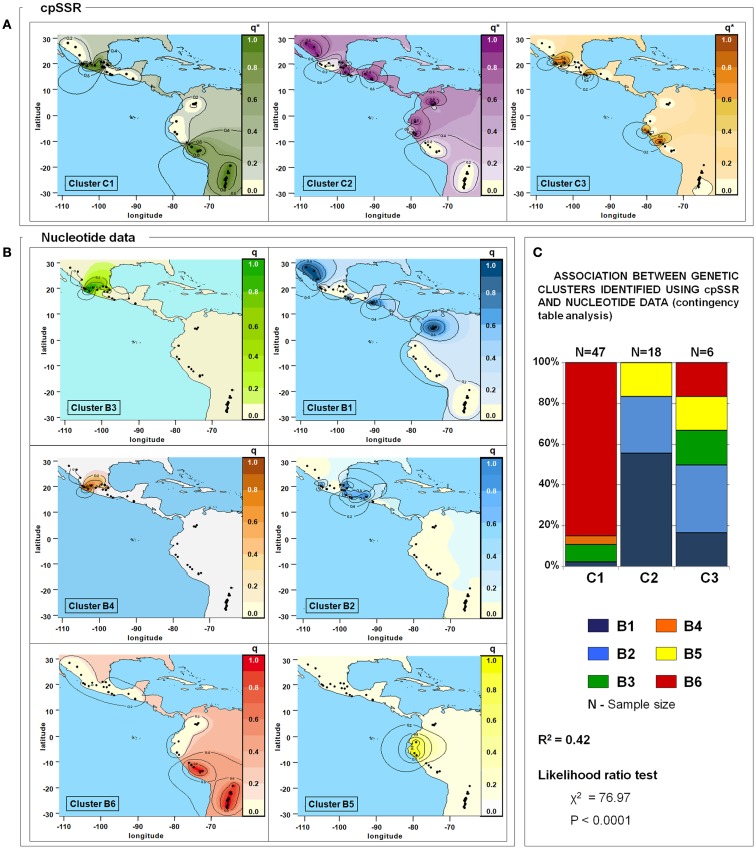
**Spatial interpolation of the membership coefficients (*q*) for the clusters identified by the population structure analysis using cpSSR (A) and for the nucleotide data (B), and results of the association test between these (C)**. q*, for cpSSRs, the geographical representation of the membership coefficients represents an approximation to easily compare the results obtained for the two different markers; indeed cpSSR q* values are represented by one or zero (i.e., membership or non-membership to one cluster), even if the spatial interpolation gives intermediate values. Only the 71 accessions shared between this study and that of Bitocchi et al. (2012) are included in this analysis. Latitude and longitude are expressed in the Universal Transverse Mercator system.

The availability of sequence data for five gene fragments for 71 out of the 109 *P. vulgaris* accessions allowed a comparison between these different kinds of data (plastidial and nuclear). Three clusters were identified by the analysis carried out with cpSSRs. They corresponded to clusters (*C*1, *C*2, and *C*3) determined previously using all the accessions, while the Cluster *C*4 was not determined due to the exclusion, in this comparative analysis, of the *P. coccineus* accessions. Six clusters (*B*1, *B*2, *B*3, *B*4, *B*5, and *B*6), as in Bitocchi et al. (2012) were identified with nuclear nucleotide data. The distribution of the accessions into the nucleotide data and cpSSR clusters is reported in Table 6. Figures 5A,B shows the geographical distribution of these clusters. The analysis of contingency tables indicated a significant association (*P* < 0.0001; likelihood ratio χ^2^ test) between the genetic clusters obtained with these different markers (Figure 5C). In particular, cluster *C*1 was represented by clusters *B*3, *B*4, and *B*6, while cluster *C*2 included the *B*1, *B*2, and *B*5 clusters. In contrast, cluster *C*3 did not show any associations, although it is represented by accessions from the gene pools from Mesoamerica (*B*1, *B*2, *B*3), the Andes (*B*6), and northern Peru and Ecuador (*B*5).

**Table 6:** **Distribution of the 71 accessions shared between nucleotide and cpSSR data into the six nucleotide data clusters (*B*1, *B*2, *B*3, *B*4, *B*5, and *B*6) and the four cpSSR clusters (*C*1, *C*2, *C*3, *C*4) identified by the BAPS analysis**.

Accession	cpSSR cluster			Nucleotide data cluster					
	*C*1	*C*2	*C*3	*B*1	*B*2	*B*3	*B*4	*B*5	*B*6
MW	7	15	4	12	7	5	2	–	–
AW	40	–	1	–	–	–	–	–	41
PhI	–	3	1	–	–	–	–	4	–
Overall	47	18	6	12	7	5	2	4	41

*MW, Mesoamerican wild; AW, Andean wild; PhI, northern Peru and Ecuador*.

## Discussion

The main aim of the present study was to investigate the complex evolutionary history that characterizes *P. vulgaris* through an analysis of its genetic diversity at the plastidial DNA level, in comparison with the study of Bitocchi et al. (2012) that was based on nuclear nucleotide data. Thus, taking into account the specific characteristics of the plastidial genome (haploidy, lack of recombination, uniparental inheritance), we used cpSSRs to contribute to the existing knowledge of the evolution of the common bean and its closely related species, and to provide new insights, especially considering that comparisons of data obtained through analyses of nuclear and cytoplasmic DNA can provide contrasting information on evolutionary processes (Birky, 1988; McCauley, 1995; Ennos et al., 1999; Provan et al., 1999; Weising and Gardner, 1999; Ishii et al., 2001; Lira et al., 2003; Ueno et al., 2005).

The data obtained here are in agreement with the Mesoamerican origin of *P. vulgaris*, thus confirming the findings of Bitocchi et al. (2012), where the nucleotide diversity at five nuclear gene fragments in a wide sample of wild *P. vulgaris* accessions was analyzed (mostly shared with the present study). Moreover, the absence of phaseolin type I in the Mesoamerican gene pool might be due to its extinction in Mesoamerica, or it might still be present, but just not included in the samples analyzed in the literature.

The first outcome was the reduction in the genetic diversity (26%) in the Andean gene pool, compared to that of Mesoamerica. This has already been shown, even if to different extents, by analyses carried out with different nuclear molecular markers (SSRs: 7%, Kwak and Gepts, 2009; AFLPs: 45%, Rossi et al., 2009) and sequence data (90%, Bitocchi et al., 2012). In particular, the loss of diversity detected with cpSSRs is intermediate between the SSRs and AFLPs, as is their mutation rate (10^−3^–10^−5^ mutations per generation; Provan et al., 1999; Marshall et al., 2002). Indeed SSRs are characterized by a very high mutation rate (10^−3^–10^−4^ mutations per generation; Estoup and Angers, 1998; Mariette et al., 2001; Udupa and Baum, 2001; Vigouroux et al., 2002; Thuillet et al., 2005; Garoia et al., 2007) and AFLPs by a lower one (10^−6^–10^−5^ mutations per generation; Mariette et al., 2001; Gaudeul et al., 2004; Kropf et al., 2009). Consistent with the evidence obtained for the nuclear genome (Kwak and Gepts, 2009; Rossi et al., 2009; Nanni et al., 2011; Bitocchi et al., 2012), our data provide further evidence of the bottleneck that occurred before domestication of the common bean in the Andes, which led to impoverishment of the genetic diversity also at the plastidial level in the present gene pool. Moreover, this confirms the strong relationship between the mutation rate and the time needed for a population to recover the genetic diversity that can be lost after a bottleneck: the higher the mutation rate, the shorter the time needed (Glémin and Bataillon, 2009; Rossi et al., 2009; Nanni et al., 2011; Bitocchi et al., 2012, 2013).

Moreover, the BAPS analysis allows the division into three main clusters for the *P. vulgaris* accessions (*C*1, *C*2, *C*3). The Andean accessions are almost all included in cluster *C*1, with the only exception being an accession from southern Peru that belongs to cluster *C*3. Considering the nuclear data, cluster *C*1 is significantly associated with clusters *B*3, *B*6, and *B*4. This supports the close relationship between the Andean (*B*6) and the MW accessions from central Mexico (*B*3; Bitocchi et al., 2012), which indicates that these MW accessions represent the most probable plant material that spread and adapted to the southern part of the Andes.

Cluster *C*2 is characterized by the Mesoamerican accessions assigned using nucleotide data to clusters *B*1 and *B*2, and three of the seven PhI accessions, while cluster *C*3 groups the accessions that are representative of all of the gene pools (Mesoamerican, Andean, and northern Peru and Ecuador). These data provide further confirmation of the evidence highlighted by the nuclear data (Bitocchi et al., 2012); indeed, the Mesoamerican population is highly subdivided also at the plastidial level, and all of the genetic groups identified are present in particular in Central Mexico, which indicates this geographical area as the center of origin of *P. vulgaris*.

However, an interesting and novel outcome is revealed by the cpSSRs, which is probably due to the different characteristics of the nuclear and plastidial genome (and in particular to the presence of recombination for the nuclear genome): the identification of cluster *C*3 as a genetic group that incorporates accessions that are representative of all of the gene pools (MW, AW, PhI) and are not significantly associated with any genetic cluster identified with the nuclear data. In particular, almost all of the MW in cluster *C*3 are from Central Mexico, with the only exception being one Colombian genotype; moreover, cluster *C*3 comprised four PhI accessions and one AW accession. The wide distribution in cluster *C*3 can be interpreted as evidence that these accessions carry the ancestral plastidial genome that spread over the entire distribution that is now covered by *P. vulgaris*. This pattern is also confirmed by the *R*_ST_ divergence estimations, where cluster *C*3 shows the lowest values compared to all of the other clusters, including most of the various alleles, when the size of the alleles is considered as a measure of the evolutionary distance among alleles. However, the same does not hold when the infinite allele model is considered: *F*_ST_. Indeed, for *F*_ST_, *C*2 shows the lowest divergence. This appears to be determined by the higher diversity (He) of *C*2 compared to *C*3, but not as alleles number (richness), with *C*2 showing the more uniform distribution of allele frequencies. Thus, we can speculate that the different results obtained for *R*_ST_ and *F*_ST_ might be the result of the more precise estimation of allele divergence using *R*_ST_ and because *C*3 has more skewed allele frequencies due to the drift (e.g., a bottleneck).

The membership of the two *P. coccineus* genotypes to cluster *C*2 suggests that this cluster can be considered as having been derived from an ancestral lineage from which *P. vulgaris* separated from *P. coccineus*. Alternatively, this might result from post speciation introgression from *P. vulgaris* (with *P. vulgaris* as the maternal parent of the initial hybridization). This putative introgression of plastidial DNA from *P. vulgaris* to *P. coccineus* is consistent with the hypothesis that the *P. dumosus* species originated from a cross of *P. vulgaris* as maternal and *P. coccineus* as paternal parent, followed by successive backcrosses from *P. coccineus* as paternal donor (Schmit et al., 1993; Llaca et al., 1994; Angioi et al., 2009a). Indeed, *P. dumosus* is closer to *P. coccineus* according to nuclear DNA comparisons (Piñero and Eguiarte, 1988; Delgado-Salinas et al., 1999), while according to chloroplast DNA comparisons it appears to be more closely related to *P. vulgaris* (Llaca et al., 1994; Angioi et al., 2009a). These outcomes reveal the complexity of the evolution of *P. vulgaris* within the evolutionary history of its closely related species, *P. coccineus* and *P. dumosus* (Schmit et al., 1993; Delgado-Salinas et al., 1999, 2006; Chacón et al., 2007), both of which are found in Mesoamerica (Schmit and Debouck, 1991; Freytag and Debouck, 2002). In spite of the marked differences in mating systems and life cycles, *P. coccineus* (predominantly allogamous and perennial), *P. vulgaris* (predominantly autogamous and annual), and *P. dumosus* (intermediate characteristics between *P. coccineus* and *P. vulgaris*) are partially intercrossable, although only when *P. vulgaris* is the female parent (Mendel, 1866; Wall, 1970; Shii et al., 1982; Hucl and Scoles, 1985). However, further studies should be carried out here, to compare a larger sample that includes genotypes from all three of these sister species and uses both nuclear and plastidial DNA analyses.

## Conclusion

Chloroplast SSRs are widely used for evolutionary and phylogenetic studies as they have been demonstrated to be effective indicators of the genetic structure of a population. Therefore, we used this alternative form of analysis (with respect to nuclear data) with the aim of obtaining a more detailed picture of the history of the common bean. These cpSSR data strongly support the nuclear data of Bitocchi et al. (2012), that indicated a clear Mesoamerican origin of this species, and in particular, they support Central Mexico as, with high probability, the cradle of common bean diversity.

A novel outcome was also provided by these analyses based on the polymorphism at the chloroplast DNA level: the identification of a genetic group (cluster *C*3) that includes accessions distributed from northern Mexico to Peru that appear to carry a putative ancestral plastidial genome.

Finally, the present study highlights the potential to evaluate the evolutionary history of *P. vulgaris* within the evolution of the whole species complex that includes *P. vulgaris*, *P. coccineus*, and *P. dumosus*. A deeper study of the formation and evolution of these closely related and intercrossable species will be intriguing from an evolutionary point of view. At the same time, such data should be particularly relevant for common bean breeding programs, as demonstrated by the increasing interest in the development of interspecific lines (*P. vulgaris-P. coccineus* and *P. vulgaris*-*P. dumosus* crosses) for the introgression of important traits; e.g., resistance to biotic and abiotic stress in *P. vulgaris* elite germplasm (Singh et al., 2009; Klaedtke et al., 2012).

## Conflict of Interest Statement

The authors declare that the research was conducted in the absence of any commercial or financial relationships that could be construed as a potential conflict of interest.

## Appendix

**Table A1:** **List of accessions used in this study**.

Accession code^1^	Synonyms	Species	Donor^2^	Pop code^3^	Country	Department, state, or province	Latitude	Longitude	BAPS cluster (cpSSR)	BAPS cluster (nucleotide data); *q* ≥ 0.6^4^
G21113	LEROI COL-14, NI-922	*P. vulgaris*	CIAT	MW	Colombia	Cundinamarca	44,833	−73,933	*C*2	/
G22304	LEROI COL-13, NI-1142	*P. vulgaris*	CIAT	MW	Colombia	Cundinamarca	44,833	−73,933	*C*3	/
G21115•	LEROI COL-23, NI-926	*P. vulgaris*	CIAT	MW	Colombia	Cundinamarca	45,333	−73,917	*C*2	*B*1
G21117•	LEROI COL-28, NI-937	*P. vulgaris*	CIAT	MW	Colombia	Cundinamarca	46,667	−74.4	*C*2	*B*1
G22303•	LEROI COL-22, NI-1141	*P. vulgaris*	CIAT	MW	Colombia	Cundinamarca	45,333	−73,917	C1	*B*1
G23462•	LEROI COL-15, NI-1256, X-636	*P. vulgaris*	CIAT	MW	Colombia	Cundinamarca	50,833	−73,617	C2	*B*1
G2771	GENTRY 22274; PI318702	*P. vulgaris*	CIAT	MW	Mexico	Nayarit	211,667	−104.37	*C*3	/
G11051	DGD-451	*P. vulgaris*	CIAT	MW	Mexico	Jalisco	207,667	−103.4	*C*3	/
G12927	M7278-G, PI417689	*P. vulgaris*	CIAT	MW	Mexico	Jalisco	20.7	−102.35	*C*3	/
G12957	M7424-C, PI417786	*P. vulgaris*	CIAT	MW	Mexico	Jalisco	20.9	−102.37	*C*1	/
G23418	DGD-2111	*P. vulgaris*	CIAT	MW	Costa Rica	San Jose	98,667	−84,117	*C*2	/
G23558	OAXACA 112	*P. vulgaris*	CIAT	MW	Mexico	Oaxaca	163,333	−95,233	*C*2	/
G24366	JSG & LOS-150	*P. vulgaris*	CIAT	MW	Mexico	Jalisco	204,833	−103.4	*C*1	/
PI417775	G12949, M7408-P	*P. vulgaris*	USDA	MW	Mexico	Jalisco	20.64	−102.41	*C*3	/
W612107	CR-93-004	*P. vulgaris*	USDA	MW	Costa Rica	Puntarenas	8.95	−83,038	*C*2	/
G9989•	HM7395-BULK	*P. vulgaris*	CIAT	MW	Mexico	Jalisco	20.5	−104.82	*C*3	*B*1
G19906•	DGD-1610	*P. vulgaris*	CIAT	MW	Guatemala	Sacatepequez	14.45	−90.7	*C*2	*B*1
G19907•	DGD-1611	*P. vulgaris*	CIAT	MW	Guatemala	Sacatepequez	14.45	−90,817	*C*2	*B*1
G19909•	DGD-1619	*P. vulgaris*	CIAT	MW	Guatemala	Sacatepequez	14.55	−90,833	*C*2	*B*1
G22837•	GN 84127/BB 8480, P16-001	*P. vulgaris*	CIAT	MW	Mexico	Chihuahua	269,333	−106.42	*C*2	*B*1
G23463•	GN 84154, L 625	*P. vulgaris*	CIAT	MW	Mexico	Chihuahua	283,333	−108.5	*C*2	*B*1
G24378•	JSG & LOS-199	*P. vulgaris*	CIAT	MW	Mexico	Oaxaca	16.4	−97,083	*C*2	*B*1
G50899•	LEROI MEX-26, NI-1144	*P. vulgaris*	CIAT	MW	Mexico	Durango	237,833	−105.37	*C*2	*B*1
G11056•	DGD-490	*P. vulgaris*	CIAT	MW	Mexico	Jalisco	205,667	−104.77	*C*3	*B*2
G20515•	M8137B-1	*P. vulgaris*	CIAT	MW	Mexico	Puebla	19.8	−97,783	*C*2	*B*2
G23429•	DGD-2325	*P. vulgaris*	CIAT	MW	Mexico	Puebla	189,667	−98,383	*C*2	*B*2
G24571•	JSMM-4002	*P. vulgaris*	CIAT	MW	Mexico	Oaxaca	171,667	−97,983	*C*2	*B*2
G24572•	JSMM-4006	*P. vulgaris*	CIAT	MW	Mexico	Oaxaca	159,833	−96,517	*C*3	*B*2
G24599•	JAG-180	*P. vulgaris*	CIAT	MW	Mexico	Chiapas	164,833	−92,517	*C*2	*B*2
G50415•	JAG-209	*P. vulgaris*	CIAT	MW	Mexico	Hidalgo	20.85	−98,717	*C*2	*B*2
G11050•	DGD-439	*P. vulgaris*	CIAT	MW	Mexico	Michoacan	196,833	−101.27	*C*1	*B*4
G12922•	M7278-A, PI417683	*P. vulgaris*	CIAT	MW	Mexico	Jalisco	20.7	−102.35	*C*3	*B*3
G12979•	M7439T	*P. vulgaris*	CIAT	MW	Mexico	Jalisco	201,167	−104.37	*C*1	*B*3
G23415A•	DGD-2077	*P. vulgaris*	CIAT	MW	Mexico	Queretaro	211,333	−99,617	*C*1	*B*3
G23652•	M2058	*P. vulgaris*	CIAT	MW	Mexico	Puebla	19.8	−97,783	*C*1	*B*3
G12865•	GENTRY 22199, PI318696	*P. vulgaris*	CIAT	MW	Mexico	Jalisco	193,333	−103.25	*C*1	*B*3
G12873•	PI325678, GENTRY22492	*P. vulgaris*	CIAT	MW	Mexico	Morelos	19	−99.25	*C*1	*B*4
G10012	MORELOS 646, V-1434	*P. vulgaris*	CIAT	MW	Mexico	Morelos	188,833	−99.15	*C*2	/
G12872	GENTRY 22404, PI325677	*P. vulgaris*	CIAT	MW	Mexico	Morelos	189,667	−99.1	*C*1	/
G12877	GENTRY 22530, PI325683	*P. vulgaris*	CIAT	MW	Mexico	Morelos	18.95	−99,217	C3	/
G12896	M7230, PI417629	*P. vulgaris*	CIAT	MW	Mexico	Michoacan	201,333	−102.08	*C*3	/
G12924	M7278-C, PI417685	*P. vulgaris*	CIAT	MW	Mexico	Jalisco	20.7	−102.35	*C*3	/
G12930	M7278-L, PI417692	*P. vulgaris*	CIAT	MW	Mexico	Jalisco	20.7	−102.35	*C*3	/
G13018	MORELOS 654, V-1438	*P. vulgaris*	CIAT	MW	Mexico	Morelos	188,833	−99.15	*C*1	/
G13505	MORELOS 635, NI-404	*P. vulgaris*	CIAT	MW	Mexico	Morelos	188,833	−99.15	*C*2	/
G12866	GENTRY 22202, PI318697	*P. vulgaris*	USDA	MW	Mexico	Jalisco	19,683	−103.48	*C*1	/
CHWENN2		*P. vulgaris*	UNIVPM	MW	Mexico	Chiapas	164,833	−92,517	*C*3	/
CHWETE16		*P. vulgaris*	UNIVPM	MW	Mexico	Chiapas	164,833	−92,517	*C*3	/
DGW15		*P. vulgaris*	UNIVPM	MW	Mexico	Durango	237,833	−105.37	*C*3	/
111d		*P. vulgaris*	UNIVPM	MW	Mexico	Chiapas	164,833	−92,517	*C*3	/
JAL97		*P. vulgaris*	UNIVPM	MW	Mexico	Jalisco	204,833	−103.4	*C*3	/
MOW5		*P. vulgaris*	UNIVPM	MW	Mexico	Morelos	189,667	−99.1	*C*3	/
MXW17		*P. vulgaris*	UNIVPM	MW	Mexico	–	***	***	*C*3	/
PUW21		*P. vulgaris*	UNIVPM	MW	Mexico	Puebla	***	***	*C*3	/
G23415	DGD-2077	*P. vulgaris*	CIAT	MW	Mexico	Queretaro	211,333	−99,617	*C*1	/
G23423C	DGD-2157	*P. vulgaris*	CIAT	AW	Perù	Apurimac	−13.85	−72,967	*C*1	/
W617481	PI638874	*P. vulgaris*	USDA	AW	Argentina	Jujuy	−22,267	−64,683	*C*1	/
W617500	PI640966	*P. vulgaris*	USDA	AW	Argentina	Salta	−24.65	−65,367	*C*1	/
W617501	PI640967	*P. vulgaris*	USDA	AW	Argentina	Salta	−24.65	−65,367	*C*1	/
G7225	APURIMAC 76	*P. vulgaris*	CIAT	AW	Perù	Apurimac	−13,667	−72,883	*C*1	/
W617467	PI638865	*P. vulgaris*	USDA	AW	Argentina	Tucuman	−26,217	−65,527	*C*1	/
G7469•	NI-029	*P. vulgaris*	CIAT	AW	Argentina	***	***	***	*C*1	*B*6
G10024•	NI-190	*P. vulgaris*	CIAT	AW	Argentina	***	***	***	*C*1	*B*6
G12856•	PI260405, SMITH PV-1	*P. vulgaris*	CIAT	AW	Perù	Huanuco	−10,333	−76,183	*C*3	*B*6
G19888•	DGD-623	*P. vulgaris*	CIAT	AW	Argentina	Jujuy	−24,167	−65.6	*C*1	*B*6
G19889•	DGD-624	*P. vulgaris*	CIAT	AW	Argentina	Jujuy	−24.25	−65,283	*C*1	*B*6
G19891•	DGD-628	*P. vulgaris*	CIAT	AW	Argentina	Salta	−25,117	−65,617	*C*1	*B*6
G19892•	DGD-629	*P. vulgaris*	CIAT	AW	Argentina	Salta	−25.15	−65.65	*C*1	*B*6
G19893•	DGD-630, NEEMA S-211/S-226	*P. vulgaris*	CIAT	AW	Argentina	Salta	−24,633	−65,483	*C*1	*B*6
G19895•	DGD-637, NEEMA T-711/T-717	*P. vulgaris*	CIAT	AW	Argentina	Tucuman	−26,433	−65,517	*C*1	*B*6
G19896•	DGD-639	*P. vulgaris*	CIAT	AW	Argentina	Tucuman	−26,217	−65,583	*C*1	*B*6
G19897•	DGD-643, NEEMA T-911/T-917	*P. vulgaris*	CIAT	AW	Argentina	Tucuman	−27,317	−65,917	*C*1	*B*6
G19898•	DGD-644	*P. vulgaris*	CIAT	AW	Argentina	Tucuman	−27,333	−65.95	*C*1	*B*6
G19901•	DGD-649	*P. vulgaris*	CIAT	AW	Argentina	Tucuman	−26,933	−65.7	*C*1	*B*6
G21194•	DGD-621	*P. vulgaris*	CIAT	AW	Argentina	Jujuy	−24,117	−65,417	*C*1	*B*6
G21197•	DGD-1711	*P. vulgaris*	CIAT	AW	Argentina	Jujuy	−24.05	−65.45	*C*1	*B*6
G21198•	DGD-1712	*P. vulgaris*	CIAT	AW	Argentina	Jujuy	−24,067	−65,367	*C*1	*B*6
G21199•	DGD-1713	*P. vulgaris*	CIAT	AW	Argentina	Jujuy	−23,917	−65.35	*C*1	*B*6
G21201•	DGD-1716	*P. vulgaris*	CIAT	AW	Argentina	Salta	−22.25	−65	*C*1	*B*6
G23420•	DGD-2147	*P. vulgaris*	CIAT	AW	Perù	Junin	−11.2	−75,483	*C*1	*B*6
G23421•	DGD-2152	*P. vulgaris*	CIAT	AW	Perù	Junin	−12,017	−74,883	*C*1	*B*6
G23422•	DGD-2156	*P. vulgaris*	CIAT	AW	Perù	Apurimac	−14	−73,167	*C*1	*B*6
G23426•	DGD-2295	*P. vulgaris*	CIAT	AW	Perù	Apurimac	−13,617	−73.2	*C*1	*B*6
G23444•	DGD-2497	*P. vulgaris*	CIAT	AW	Bolivia	Chuquisaca	−19.3	−64,317	*C*1	*B*6
G23445•	DGD-2501	*P. vulgaris*	CIAT	AW	Bolivia	Tarija	−21,533	−64,867	*C*1	*B*6
G23455•	DGD-2581	*P. vulgaris*	CIAT	AW	Perù	Cuzco	−13.5	−72,483	*C*1	*B*6
W617466•	PI638864	*P. vulgaris*	USDA	AW	Argentina	Tucuman	−26,233	−65,483	*C*1	*B*6
W617468•	PI638866	*P. vulgaris*	USDA	AW	Argentina	Tucuman	−27,817	−65,783	*C*1	*B*6
W617469•	PI638867	*P. vulgaris*	USDA	AW	Argentina	Tucuman	−27,797	−65,785	*C*1	*B*6
W617470•	PI640964	*P. vulgaris*	USDA	AW	Argentina	Tucuman	−26,383	−65,467	*C*1	*B*6
W617471•	PI638868	*P. vulgaris*	USDA	AW	Argentina	Tucuman	−26,383	−65,533	*C*1	*B*6
W617472•	PI638869	*P. vulgaris*	USDA	AW	Argentina	Tucuman	−26.95	−65.7	*C*1	*B*6
W617473•	PI638870	*P. vulgaris*	USDA	AW	Argentina	Salta	−26.1	−65.6	*C*1	*B*6
W617474•	PI640965	*P. vulgaris*	USDA	AW	Argentina	Salta	−25,161	−65,611	*C*1	*B*6
W617475•	PI638871	*P. vulgaris*	USDA	AW	Argentina	Salta	−25,167	−65,617	*C*1	*B*6
W617476•	PI638872	*P. vulgaris*	USDA	AW	Argentina	Salta	−25,166	−65,649	*C*1	*B*6
W617478•	PI638873	*P. vulgaris*	USDA	AW	Argentina	Salta	−24,896	−65,801	*C*1	*B*6
W617486•	PI638875	*P. vulgaris*	USDA	AW	Argentina	Jujuy	−22,267	−64,683	*C*1	*B*6
W617499•	PI661807	*P. vulgaris*	USDA	AW	Argentina	Salta	−24.9	−65,483	*C*1	*B*6
W617502•	PI640968	*P. vulgaris*	USDA	AW	Argentina	Salta	−24,717	−65,483	*C*1	*B*6
W618821•	PI638897, DGD3038	*P. vulgaris*	USDA	AW	Bolivia	Chuquisaca	−19,283	−64,333	*C*1	*B*6
W618826•	PI638898, DGD3044	*P. vulgaris*	USDA	AW	Bolivia	Chuquisaca	−19,283	−64,333	*C*1	*B*6
G23581	DGD-2765	*P. vulgaris*	CIAT	PhI	Ecuador	Azuay	−3.2	−79,183	*C*3	/
G23582	DGD-2769	*P. vulgaris*	CIAT	PhI	Ecuador	Chimborazo	−22,667	−78,967	*C*3	/
G23724	DGD-2881, PI557544, W6 8245	*P. vulgaris*	CIAT	PhI	Ecuador	Loja	−43,167	−79,933	*C*3	/
G21245•	DGD-1962	*P. vulgaris*	CIAT	PhI	Perù	Cajamarca	−71,167	−78,783	*C*2	*B*5
G23585•	DGD-2855	*P. vulgaris*	CIAT	PhI	Perù	Cajamarca	−6.35	−79.4	*C*2	*B*5
G23587•	DGD-2858	*P. vulgaris*	CIAT	PhI	Perù	Cajamarca	−6.35	−79.4	*C*3	*B*5
G23726•	DGD-2889	*P. vulgaris*	CIAT	PhI	Ecuador	Chimborazo	−19,667	−78.95	*C*2	*B*5
PI535280	TARS212, 78-G-4	*P. coccineus*	USDA	–	Guatemala	Sacatepequez	14.43	−90.95	*C*2	/
PI535287	TARS222, 78-G-15	*P. coccineus*	USDA	–	Guatemala	Sacatepequez	14.67	−90.75	*C*2	/
PI325584	ACAHUATE	*P. coccineus*	USDA	–	Mexico	Puebla	19,816	−978,166	*C*4	/
PI417608	M7417-G	*P. coccineus*	USDA	–	Mexico	Jalisco	20,866	−102,367	*C*4	/
PI417611	M7423-A	*P. coccineus*	USDA	–	Mexico	Jalisco	20,866	−102,366	*C*4	/
PI417592	M7399-V	*P. coccineus*	USDA	–	Mexico	Jalisco	25.56	−106.37	*C*4	/
PI430191	M7402-U	*P. coccineus*	USDA	–	Mexico	Chihuahua	28.6	−107,167	*C*4	/
PI430192	M7402-V	*P. coccineus*	USDA	–	Mexico	Chihuahua	28.6	−107,167	*C*4	/
CX 03		*P. coccineus*	UNIVPM	–	Mexico	Morelos	***	***	*C*4	/
CF19		*P. coccineus*	UNIVPM	–	Mexico	Morelos	***	***	*C*4	/

*^1^Population code: WM, wild Mesoamerican; WA, wild Andean; PhI, Phaseolin I type*.

*^2^•(dot) indicates the *P. vulgaris* accessions shared with the study of Bitocchi et al. (2012), showing high-quality sequences for all of the five Leg markers analyzed in Bitocchi et al. (2012); these accessions were used to compare the population structure results obtained using both cpSSR and nucleotide data*.

*^3^CIAT, International Centre for Tropical Agriculture; USDA, United States Department of Agriculture; UNIVPM, Università Politecnica delle Marche*.

*^4^*q*, percentage of membership to one of the six clusters identified using nucleotide data; a *q* threshold value of 0.6 was considered to assign accessions to clusters*.

**Table A2:** **List of SSR used in this study**.

Locus	Primer sequence 5′–3′		PCR conditions^a^	Reference
ccSSR2	fw-AATCCTGGACGTGAAGAATAA	rev-AATCCCTCTCTTTCCGTTGA	1	Chung and Staub (2003)
ccSSR4	fw-AGGTTCAAATCCTATTGGACGCA	rev-TTTTGAAAGAAGCTATTCARGAAC	1	Chung and Staub (2003)
ccSSR7	fw-CGGGAAGGGCTCGKGCAG	rev-GTTCGAATCCCTCTCTCTCCTTTT	1	Chung and Staub (2003)
ccSSR8	fw-TTGATCTTTACGGTGCTTCCTCTA	rev-TCATTACGTGCGACTATCTCC	1	Chung and Staub (2003)
ccSSR9	fw-GAGGATACACGACAGARGGARTTG	rev-CCTATTACAGAGATGGTGYGATTT	1	Chung and Staub (2003)
ccSSR11	fw-TTGGCTACTCTAACCTTCCC	rev-ACCATAGAAACGAWGGAACCCACT	2	Chung and Staub (2003)
ccSSR12	fw-CCAAAAACTTGGAGATCCAACTAC	rev-TTCCATAGATTCGATCGTGGTTTA	1	Chung and Staub (2003)
ccSSR15	fw-GCTTATGACCTCCCCCTCTATGC	rev-TGCATTACAGACGTATGATCATTA	1	Chung and Staub (2003)
ccSSR16	fw-TACGAGATCACCCCTTTCATTC	rev-CCTGGCCCAACCCTAGACA	1	Chung and Staub (2003)
ccSSR18	fw-TCGTTGGATTTCTTCDGGACATTT	rev-CCCAATATCATCATACTTACRTGC	1	Chung and Staub (2003)
ccSSR19	fw-CTATGCAGCTCTTTTATGYGGATC	rev-TCCARGTAATAAATGCCCAAGTT	1	Chung and Staub (2003)
ccSSR20	fw-CCGCARATATTGGAAAAACWACAA	rev-GCTAARCAAATWGCTTCTGCTCC	1	Chung and Staub (2003)
ccmp2	fw-GATCCCGGACGTAATCCTG	rev-ATCGTACCGAGGGTTCGAAT	1	Weising and Gardner (1999)
ccmp3	fw-CAGACCAAAAGCTGACATAG	rev-GTTTCATTCGGCTCCTTTAT	3	Weising and Gardner (1999)
cp1	fw-CAAAATCAAAAGAGCGATTAGG	rev-GTCAAACCCATGAACGGACT	1	Angioi et al. (2009a)
cp2	fw-TCTGTTTTGACCATATCGCACT	rev-GTCCATAAATAGATTCCCGAAAAA	4	Angioi et al. (2009a)
cp3	fw-TCGGTGTAAATTGATAAAACGAAA	rev-TGCCTAGCAAAAGACTCTAAGAAAG	4	Angioi et al. (2009a)

*^a^PCR conditions: 1: 5 min at 94°C; 35 cycles of 1′ at 94°C 1′ at 50°C 1′ at 72°C; 35′ at 72°C; 2: 5′ at 94°C; touch down cycles 53–45°C with −1°C/2 cycles, 1′ at 72°C;20 cycles of 1′ at 94°C, 1′ at 45°C 1′ at 72°C; 35′ at 72°C; 3: 5′ at 94°C; touch down cycles 53–43°C with −1°C/2 cycles, 1′ at 72°C; 20 cycles of 1′ at 94°C 1′ at 43°C. 1′ at 72°C; 35′ at 72°C; 4: 5′ at 94°C; 30 cycles of 30 s at 94°C 30 s at 48°C 30 s at 72°C; 35′ at 72°C*.

**Table A3:** **Number of alleles (Na) and gene diversity (He, Nei, 1978) in the overall, *P. vulgaris* and *P. coccineus* samples for each of the 17 cpSSRs used**.

Locus	cp1	cp2	cp3	ccmp2	ccmp3	ccSSR2	ccSSR4	ccSSR7	ccSSR8	ccSSR9	ccSSR11	ccSSR12	ccSSR15	ccSSR16	ccSSR18	ccSSR19	ccSSR20
**OVERALL**																	
**He**	0.48	0.13	0.15	0.68	0.52	0.66	0.63	0.71	0.65	0.61	0.79	0.51	0.60	0.33	0.46	0.44	0.85
**Na**	4	2	6	4	4	4	6	7	6	4	9	3	3	3	6	3	12
Allelic range (bp)	111–114	180–183	154–171	196–199	79–94	167–170	244–249	299–308	224–265	133–136	164–183	203–206	262–264	353–355	260–266	376–378	312–324
***P. vulgaris***																	
**He**	0.43	0.00	0.02	0.67	0.48	0.65	0.63	0.66	0.64	0.60	0.76	0.44	0.61	0.30	0.49	0.46	0.84
**Na**	4	1	2	4	3	4	5	6	6	3	8	2	3	3	6	3	11
Allelic range (bp)	111–114	180	170–171	196–199	83–94	167–170	245–249	299–308	224–265	133–135	164–174	205–206	262–264	353–355	260–266	376–378	314–324
***P. coccineus***																	
**He**	0.00	0.36	0.82	0.00	0.64	0.36	0.36	0.64	0.00	0.64	0.64	0.36	0.00	0.53	0.00	0.00	0.84
**Na**	1	2	5	1	3	2	2	4	1	4	3	2	1	2	1	1	6
Alleles (bp)	112	180–183	154–170	196	79–84	167–168	244–245	303–307	260	133–136	164–183	203–206	263	353–354	263	376	312–320
